# Characterization of Iron Accumulation in Deep Gray Matter in Myotonic Dystrophy Type 1 and 2 Using Quantitative Susceptibility Mapping and R2^*^ Relaxometry: A Magnetic Resonance Imaging Study at 3 Tesla

**DOI:** 10.3389/fneur.2019.01320

**Published:** 2019-12-13

**Authors:** Sevda Ates, Andreas Deistung, Ruth Schneider, Christian Prehn, Carsten Lukas, Jürgen R. Reichenbach, Christiane Schneider-Gold, Barbara Bellenberg

**Affiliations:** ^1^Department of Neurology, St. Josef Hospital, Ruhr-University Bochum, Bochum, Germany; ^2^Department of Radiology, University Hospital Halle (Saale), Halle (Saale), Germany; ^3^Medical Physics Group, Institute of Diagnostic and Interventional Radiology, Jena University Hospital, Friedrich Schiller-University, Jena, Germany; ^4^Institute of Neuroradiology, St. Josef Hospital, Ruhr-University Bochum, Bochum, Germany; ^5^Department of Diagnostic and Interventional Radiology and Nuclear Medicine, St. Josef Hospital, Ruhr-University Bochum, Bochum, Germany

**Keywords:** myotonic dystrophy type 1, DM1, myotonic dystrophy type 2, DM2, R2^*^ relaxometry, quantitative susceptibility mapping, iron, deep gray matter

## Abstract

Quantitative mapping of the magnetic susceptibility and the effective transverse relaxation rate (R2^*^) are suitable to assess the iron content in distinct brain regions. In this prospective, explorative study the iron accumulation in deep gray matter nuclei (DGM) in myotonic dystrophy type 1 (DM1) and 2 (DM2) and its clinical and neuro-cognitive relevance using susceptibility and R2^*^ mapping was examined. Twelve classical DM1, four childhood-onset DM1 (DM1_c.o._), twelve DM2 patients and twenty-nine matched healthy controls underwent MRI at 3 Tesla, neurological and neuro-cognitive tests. Susceptibility, R2^*^ and volumes were determined for eleven DGM structures and compared between patients and controls. Twelve classical DM1, four childhood-onset DM1, and 12 DM2 patients as well as 29 matched healthy controls underwent MRI at 3 Tesla, and neurological and neuro-cognitive tests. Susceptibility, R2^*^ and volumes were determined for 11 DGM structures and compared between patients and controls. Iron accumulation in DGM reflected by R2^*^ or susceptibility was found in the putamen and accumbens of DM1 and in DM2, but was more widespread in DM1 (caudate, pallidum, hippocampus, subthalamic nucleus, thalamus, and substantia nigra). Opposed changes of R2^*^ or susceptibility were detected in caudate, putamen and accumbens in the childhood-onset DM1 patients compared to classical DM1. R2^*^ or susceptibility alterations in DGM were significantly associated with clinical symptoms including muscular weakness (DM1), daytime sleepiness (DM1), depression (DM2), and with specific cognitive deficits in DM1 and DM2.

## Introduction

Myotonic dystrophy type 1 (DM1) and 2 (DM2) are autosomal-dominantly inherited multi-systemic diseases associated with two different polyglutamine repeat expansion mutations in non-coding gene regions (1, 2). DM1 and DM2 patients show a similar but not identical spectrum of clinical symptoms including muscular weakness and wasting, myotonia, cataracts, cardiac symptoms, and CNS involvement with cognitive impairment, depression, and daytime sleepiness (3). So far, a congenital or childhood form presenting with more severe and widespread neuropsychiatric symptoms including mental retardation, attention deficit with or without hyperactivity disorder (ADHD), autism spectrum disorder and others (4, 5) has been reported in DM1 only.

Various neuroimaging methods have been applied for either assessment of structural brain abnormalities or for detecting alterations of brain metabolism in DMs.

PET and SPECT studies showed merely diffuse and widespread metabolic changes in white matter (WM) and widespread cortical gray matter (GM) affection including reduced energy metabolism in the left frontal lobe in DM1 and in frontal and temporal regions of both sides in DM1 and DM2 (6–8). In addition, PET showed reduced energy metabolism in the striatum in both DM1 and DM2, as well as in the thalamus in DM2 patients (9). Voxel-based morphometric MRI studies revealed predominant white matter involvement in all cerebral lobes, brainstem and corpus callosum in myotonic dystrophy types 1 and 2, while partly divergent findings of gray matter decrease (cortical areas, thalamus, putamen) were reported for DM1 and DM2 (3, 10). DTI studies showed involvement of association fibers throughout the whole brain, limbic system fiber tracts, callosal body and projection fibers including internal and external capsules with more pronounced global white matter involvement in DM1 than in DM2 (10). In young patients with DM1 (age 9–19) DTI revealed abnormalities in the frontal, temporal, parietal, and occipital WM correlating with impaired working memory performance (11). In adult DM1 patients bilateral disturbances in WM integrity were seen in all tracts compared to controls and correlated in particular with working memory and processing speed (12).

A previous study of our group showed ventricular enlargement and supratentorial GM and WM atrophy. Affection of limbic structures was observed in DM1 and to an even greater extent in DM2. White matter was shown to be reduced in the splenium of the corpus callosum and in left-hemispheric WM adjacent to the pre- and post-central gyrus in DM1 while in DM2, the bilateral cingulate gyrus and subgyral medio-frontal and primary somato-sensory WM was affected (3).

With regard to atrophy of distinct DGM structures, previous studies revealed partly heterogenous findings in DM1 and DM2, as summarized in a recent review (13). In DM1, GM loss was described in all lobes, in particular the frontal, parietal and occipital region, and in the middle and superior temporal gyrus, but also subcortically in thalami and basal ganglia structures (6, 10, 13, 14). Also, reduction of gray matter volume of the accumbens and ventral diencephalon could be shown by an ROI-based approach (15). In this study, sleepiness was found to be associated with reduced volume of the right pallidum and right ventral diencephalon.

Trancranial ultrasound in DM1 and also in DM2 pointed out hyperechogenicity of the substantia nigra, and hypoechogenicity of the brainstem raphe (16).

A 5 years follow-up MRI study in DM1 and DM2 revealed mainly stable findings, but there was a mild progression of GM reduction in DM1 (17).

So far, only sparse data on iron content of deep gray matter (DGM) structures in DM1 and DM2 are available. Characterization of the iron distribution in DGM using MRI is provided by measuring the bulk magnetic susceptibility or the effective transverse relaxation rate (R2^*^). Both metrics can be deduced from the same multi-echo gradient echo MRI sequence. While the phase information of the MRI signal is utilized in quantitative susceptibility mapping to estimate the bulk magnetic susceptibility of the human brain (18–23), the magnitude signal decay is fitted using an exponential model to determine R2^*^. The dependence of both metrics, magnetic susceptibility and R2^*^, on tissue iron content has been validated in human *ex vivo* studies (24–27). However, besides paramagnetic iron, other sources including myelin or calcium deposits can alter the magnetic susceptibility and R2^*^. Due to its diamagnetic properties myelin has opposing effects on the susceptibility and R2^*^. Neurodegenerative processes leading to myelin loss would decrease R2^*^ but in parallel also lead to increased tissue susceptibility. Thus, neurodegeneration would contribute additively to the effect of iron deposition in tissue susceptibility, but not in R2^*^ (28, 29). Effects of altered cerebral levels of myelin, calcium could theoretically play a role in susceptibility or R2^*^ of brain tissue. In DM1 and DM2 myelin changes due to slowly evolving demyelinating processes mainly related to formation of white matter lesions have been observed (13, 17). Furthermore, dysregulation of calcium metabolism (30, 31), e.g., due to calcium channel dysfunction, as discussed in the pathogenesis of restless legs symptoms (RLS) in DM2 (32) could potentially influence QSM and R2^*^ measures. Still, up to now an effect of abnormal calcium levels on neuronal cells has not been investigated in the DMs.

The deep gray matter nuclei (DGM) are suggested to be involved in the control and regulation of different functional brain networks which are not only related to motor control, but also to emotional, executive and associative functional circuits (33, 34). Correlation studies between neuropsychological test results and morphometric or functional brain imaging in DM1 and DM2 exhibited interrelations between alterations of the DGM and cognitive deficits, depression and daytime sleepiness in both disorders (3, 9, 35, 36).

Using resting state MRI in DM1 an increase in functional connectivity of the bilateral posterior cingulate and the left parietal default network involving the basal ganglia and DGM nuclei was demonstrated (37). In DM1 patients, increased connectivity in the left fusiform gyrus and decreased connectivity in the right striatum were interpreted to be associated with impairment in face perception and theory of mind, and schizotypal-paranoid personality traits, respectively (38).

We hypothesize, that the magnetic susceptibility and R2^*^ are altered in specific DGM nuclei in DM1 and DM2 in comparison to age-matched healthy controls and between patient subgroups, and that, due to e.g., cellular stress, increased iron content could lead to atrophy of these structures. Further, correlation analyses were performed to examine potential associations of DGM iron content or atrophy with specific clinical or neurocognitive symptoms.

## Methods

### Ethical Considerations

The local institutional ethics committee of the Medical Faculty of the Ruhr-University Bochum (registration number 3794-10) approved this study. Written informed consent according to the Declaration of Helsinki was obtained from all individuals.

### Patients and Healthy Controls

Data of 16 DM1 and 12 DM2 patients forming part of a previous study were evaluated. Patients were examined clinically and neuropsychologically as described below. Disease onset was defined as the age when the first of the main symptoms (muscle weakness, myotonia, or cataract) has been diagnosed.

The DM1 or DM2 mutation was identified by DNA analysis of peripheral blood lymphocytes (39). The number of CTG triplet repeats of the DM1 patients was retrieved from the genetic data. For technical reasons in DM2 the exact lengths of the repeat expansions in DM2 were not determined. The DM1 group was classified into two different forms according to age at onset of the first clinical manifestation (40) into 12 classical DM1 patients (DM1) including 9 patients with an adult form (onset at age 20 years or higher) and three patients with a juvenile form (age of symptom onset between 14 and 19 years) and 4 patients with childhood onset (DM1_c.o._ age of symptom onset 7–10 years). An age-matched group of 29 healthy controls was recruited as a reference group.

Table 1 summarizes the details of the demography of the study participants and the patients' clinical status.

**Table 1:** Demography of patients and controls and the clinical symptoms.

		**DM1 classical**	**DM2**	**DM1 childhood-onset**	**healthy controls**
*N*		12	12	4	29
Female/male	N	4/8	9/3	1/3	15/14
Age at MRI (years)	Mean (SD)	45 (13)	52 (6)^a^	25 (3)^a,b,c^	44 (14)
Age at disease onset (years)	Mean (SD)	29 (10)^c^	40 (7)	9 (1)^b^^,^^c^	n.a.
Disease duration (years)	Mean (SD)	16 (7)	12 (6)	16 (3)	n.a.
CTG repeats (*N*)	Median [range]	425 [75–750]	n.a.	825 [750–920]	n.a.
Daytime sleepiness ESS	Median [range]	11 [5–20]	7 [0–20]	6 [4–12]	n.a.
Muscular weakness severity grade MIRS^d^	Median [range]	4 [1–4]	3^d^ [2–3]	3 [2–3]	n.a.
Depression BDI II	Median [range]	6 [0–27]	13 [1–42]	6 [0–19]	n.a.
CNS symptoms Parkinsonian/cerebellar	*N* (%)	1 (8%)	7 (58%)	0 (0%)	n.a.

*N, number of patients or controls; SD, standard deviation; range, minimum–maximum value; n.a., not acquired; ESS, Epworth Score Daytime Sleepiness; MIRS, muscular impairment rating scale; BDI, Beck's depression inventory score; CNS, Central Nervous System*.

a,b,c Significant group differences compared to other patient group or healthy controls using univariate ANOVA between groups (DM1, DM2, DM1_c.o._, controls) and post-hoc pairwise tests adjusted for multiple comparisons using Games-Howell correction:

a Comparison with healthy controls;

b Comparison with DM1;

c Comparison with DM2;

d *MIRS adapted for DM2, has not yet been clinically validated*.

The DM1 and DM2 groups were matched with regards to disease duration and age. The age at symptom onset of the DM2 patients was significantly higher than in DM1, while the patients with childhood-onset DM1_c.o._ were significantly younger (<30 years) than the other subgroups. The median number of CTG repeats was 825 [range: 750–920] in the DM1_c.o._ group and 425 [range: 75–750] in the DM1 group in accordance with previous studies (4, 41).

### Clinical Examinations and Neuropsychological Testing

Neurological examinations assessed CNS involvement including cerebellar and brainstem symptoms, indications of pyramidal tract lesions, restless-legs syndrome (42), or Parkinson's disease (43). Additionally, the severity of daytime sleepiness was scored by the Epworth sleepiness scale. The ESS classifies values of 0–10 as “no/moderate sleepiness,” 11–18 as “sleepy,” and 19–24 as “very sleepy” (44). Muscular involvement was quantified using the muscular impairment rating score (MIRS) which is a five-point rating scale developed to characterize the distal to proximal progression of muscular involvement in DM1 (45). The MIRS was adapted for DM2 where a proximal-to-distal type of progression is typical (46): grade 1 (no muscular impairment), 2 (minimal signs including myotonia, neck flexor weakness, no proximal weakness), 3 (proximal weakness but no distal weakness except for thumb and deep finger flexor weakness), 4 (proximal and distal weakness), and grade 5 (severe proximal and distal weakness).

### Neuropsychological Testing

The neuropsychological test batteries accounted for attention and alertness, memory and executive functions. In detail, testing for neurocognitive performance included tests for selective attention: d2-Test and Go/no-Go test from the computerized Test for Attentional Performance TAP (available at https://www.psytest.net/index.php?page=TAP-2-2&hl=en_US), and divided attention from TAP, simple reaction ability (tonic and phasic alertness from TAP), non-verbal intellectual ability [from the intelligence test LPS-Subtest 3 (47)], flexibility of thinking as a form of executive function [Regensburg verbal fluency test RWT (48)], verbal short-term memory [digitspan forward and backward from Wechsler-Memory Scale (WMS–R) (49)], non-verbal short term memory (blocktapping forward and backward from WMS-R), verbal memory RAVLT (Rey auditory verbal learning test, Version A), spatial visualization ability LPS (Leistungsprüfungssystem, LPS subtest 7), a general intelligence test (multiple choice vocabulary and a dementia screening using the clock-drawing test.

The neuropsychological findings were classified as pathological or non-pathological according to normative data using a threshold determined by one standard deviation below the normative mean. The sources of normative data for the neuropsychological tests were defined in the statistical manuals of the tests. Table 2 shows the number and percentage of patients with abnormal neuropsychological test results for those tests in which at least 25% of patients within one subgroup were affected (alertness, attention, executive functions, short-term memory).

**Table 2:** Neuropsychological test results for DM1, DM2, and DM1_c.o._: number and percentage of patients with abnormal test results if at least 25% within one subgroup were affected.

**Neuropsychological tests**		**DM1 *N* = 12**	**DM2 *N* = 12**	**DM1 _**c.o.**_*N* = 4**
Tonic alterness^a^		7 (58%)	7 (58%)	2 (50%)
Phasic alterness^a^		7 (58%)	5 (42%)	1 (25%)
Divided attention^a^	Reaction time	9 (75%)	5 (42%)	2 (50%)
	Missed events	2 (17%)	4(33%)	0 (0%)
Executive functions: alternating category verbal fluency tasks^b^	Fluency	4 (33%)	2 (17%)	1 (25%)
	Change of categories	8 (67%)	5 (42%)	1 (25%)
Verbal Short Term Memory (digitspan/WMS-R)^c^	Numbers forward	3(25%)	3 (25%)	1 (25%)
	Numbers backward	6 (50%)	4 (33%)	1 (25%)
Non-verbal short term memory (Block tapping/WMS-R)^c^	Forward	7 (58%)	8 (67%)	0 (0%)
	Backward	7 (58%)	7 (58%)	1 (25%)

*DM1_c.o._ childhood onset DM1*.

a *TAP, Test for Attentional Performance*.

b *RWT, Regensburg verbal fluency test*.

c *WMS, Regensburg verbal fluency test*.

In addition, depression was scored using the BDI-II (Beck Depression Inventory) (43) which classifies values of 0–11: normal, 11–19: mild depression, 20–26: median grade depression, >26: marked symptoms of depression.

### MRI Data Acquisition

All patients and healthy controls underwent MRI at the same 3 Tesla scanner (Philips Achieva TX, Best, The Netherlands) using a 32-channel phased-array head coil. The MRI protocol included a multi-echo 3D fast field echo sequence (repetition time [TR]: 29 ms; echo time [TE] 1: 4.6 ms; TE2: 12.6 ms; TE3: 20.6 ms; flip angle [FA]: 18°; partial parallel imaging using SENSE: 1.7; field of view [FOV]: 240 × 176; acquired voxel size: 1 × 1 × 1.2 mm interpolated to 0.5 × 0.5 × 1.2 mm; 120 axial slices; acquisition time [TA]: 6:21 min:s) for QSM and R2^*^ mapping, an isotropic T1-weighted 3D sequence (T1 fast field echo; 180 sagittal slices; FOV: 240 × 240 mm; voxel size: 1 × 1 × 1 mm; TR/TE/inversion time [TI]: 10/4.6/1,000 ms; FA: 8°, turbo factor: 164; TA: 6:00 min:s) for brain volumetry and subcortical gray matter segmentation, as well as an isotropic 3D fluid attenuated inversion recovery (FLAIR) sequence (170 sagittal slices; FOV: 240 × 240 mm; voxel size: 1 × 1 × 1 mm, TR/TE/TI: 4,800/286/1,650 ms, turbo factor: 182, TA: 6:30 min:s) for lesion quantification.

### Susceptibility and R2^*^ Mapping

Susceptibility and R2^*^ maps were reconstructed from the fast field echo data with previously described algorithms. Briefly, phase images from the three echoes were used to calculate a frequency map (50). Afterwards, frequency contributions from magnetic sources outside of the brain were removed using V-SHARP (51, 52) employing 10 radial kernels ranging between 0.5 and 5 mm radius and a truncated singular value decomposition (TSVD) threshold of 0.05. Finally, the V-SHARP corrected frequency maps were converted into quantitative susceptibility maps using homogeneity enabled incremental dipole inversion (HEIDI) (53). Susceptibility values were intrinsically referenced to the mean susceptibility of the whole brain tissue (unit: ppm parts per million). The R2^*^ maps (unit: s^−1^) were computed from the multi-echo fast field echo magnitude data by fitting the squared magnitude signal decay with logarithmic calculus (54, 55). While both R2^*^ and susceptibility are increased by iron accumulation in brain tissue, myelin has the opposite effect on susceptibility due to its diamagnetic properties. Thus, demyelination and concomitant iron increase can have a compensatory effect on R2^*^, but not on the susceptibility.

**Figure 1:**
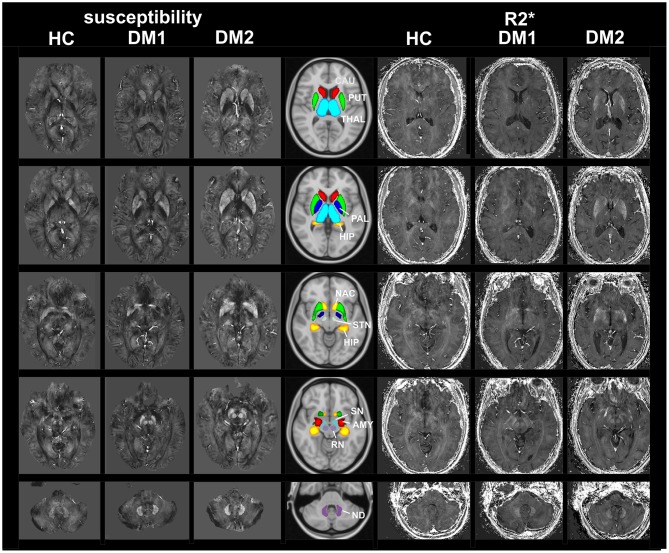
MRI based imaging techniques of the brain. Examples of susceptibility maps (three left columns) and R2^*^ maps (three right columns) illustrating marked disease and age related alterations in DGM nuclei in a DM1 patient (age 50 years, female, disease duration 8 years) and a DM2 (age: 57 years, female, disease duration 8 years) patient compared to a healthy control (age: 20 years, female). The middle column depicts the locations of the 11 investigated DGM structures as colored overlays on a standard T1 template. Major bilateral signal elevations in the patients compared to the HC can be seen in the caudate, putamen, accumbens and in the red nucleus and dentate nucleus. THAL, thalamus; CAU, caudate; PUT, putamen; PAL, pallidum; HIP, hippocampus; AMY, amygdala; NAC, accumbens; SN, substantia nigra; RN, red nucleus; STN, subthalamic nucleus; ND, dentate nucleus.

Figure 1 shows examples of susceptibility maps (three left columns) and R2^*^ maps (three right columns) illustrating alterations in DGM nuclei in a DM1 patient (age 50 years, female, disease duration 8 years) and a DM2 (age: 57 years, female, disease duration 8 years) patient compared to a healthy control (age: 20 years, female). The middle column depicts the locations of the 11 investigated DGM structures as colored overlays on a standard T1 template. Major bilateral signal elevations in the patients compared to the HC can be seen in the caudate, putamen, accumbens, and in the red nucleus and dentate nucleus.

### Data Analysis

#### Deep Gray Matter Quantification

The DGM brain structures: putamen, globus pallidus, caudate nucleus, accumbens, hippocampus, and thalamus were automatically segmented using the HC-nlFIRST approach (56). HC-nlFIRST is an optimized spin-off of the FSL-FIRST segmentation tool (57) (https://fsl.fmrib.ox.ac.uk/fsl/), and uses a composite image of the T1-weighted data and the susceptibility maps as input and improved spatial non-linear normalization to the MNI space. The mid-brain DGM nuclei were segmented using ANTs (Advanced Normalization Tools, http://stnava.github.io/ANTs/). The anatomical regions of the substantia nigra, red nucleus, subthalamic nucleus and the dentate nucleus were manually identified on a mean brain template, which was generated from the T1-weighted data of the entire cohort. After non-linear registration of the template to the individual subject datasets, the anatomical DGM regions defined in the template were then transformed into the subject space, defining individual volumes of the substantia nigra, red nucleus, subthalamic nucleus, and the dentate nucleus.

Mean DGM volumes, mean R2^*^, mean magnetic susceptibility and their respective standard deviations were calculated for each segmented brain structure and averaged over the two hemispheres. The susceptibility values of DM1 and DM2 patients were referenced to the susceptibility of the cerebrospinal fluid (CSF) in the anterior horns of the lateral ventricles. The cerebrospinal fluid (CSF) is not expected to be prone to disease-related alterations. This assumption was supported by the finding that no significant differences between patients and their matched control groups were detected in DM1 and DM2 (Table 3). In the patients with childhood-onset DM1_c.o._, we found significant susceptibility differences in CSF between patients and controls, possibly due to small ventricle sizes and contamination by the choroid plexus in this group of relatively young patients (Table 3). Therefore, susceptibility was referenced relative to the whole brain susceptibility in DM1_c.o._.

**Table 3:** Spearman correlations of magnetic susceptibility, R2^*^, and DGM volumes with age for DM1, DM2, and healthy controls.

		**Susceptibility/10**^****−3****^ **ppm**			R2*/s^****−1****^			**DGM volume/ml**		
		**Healthy controls**	**DM1**	**DM2**	**Healthy controls**	**DM1**	**DM2**	**Healthy controls**	**DM1**	**DM2**
THAL	rho	0.031	0.483	0.021	**0.397**	0.455	**−0.587**	**−0.5**	−0.42	**−0.594**
	*P*	0.873	0.112	0.948	**0.033**	0.16	**0.045**	**0.006**	0.175	**0.042**
CAU	rho	0.318	**0.629**	−0.189	0.315	0.559	−0.147	**−0.628**	**−0.72**	0.063
	*P*	0.093	**0.028**	0.557	0.096	0.059	0.649	**<0.001**	**0.008**	0.846
PUT	rho	**0.592**	**0.762**	−0.182	**0.828**	0.643	−0.021	**−0.637**	−0.448	0.105
	*P*	**0.001**	**0.004**	0.572	**<0.001**	0.024	0.948	**<0.001**	0.145	0.746
PAL	rho	**0.427**	0.07	0.084	0.323	0.287	−0.224	**−0.518**	−0.364	0.007
	*P*	**0.023**	0.829	0.795	0.088	0.366	0.484	**0.004**	0.245	0.983
HIP	rho	0.032	0.252	0.028	0.02	0.028	−0.322	−0.078	0.119	0.357
	*P*	0.873	0.43	0.931	0.919	0.931	0.308	0.686	0.713	0.255
AMY	rho	0.089	0.21	−0.021	0.063	0.329	−0.336	−0.304	−0.112	−0.056
	*P*	0.645	0.513	0.948	0.747	0.297	0.286	0.109	0.729	0.863
NAC	rho	0.076	**0.741**	**−0.615**	−0.086	0.336	**−0.608**	−0.125	−0.231	−0.294
	*P*	0.711	**0.006**	**0.033**	0.658	0.286	**0.036**	0.519	0.471	0.354
SN	rho	**0.5**	0.118	0.196	**0.487**	0.224	0.315	n.a.	n.a.	n.a.
	*P*	**0.007**	0.729	0.542	**0.009**	0.484	0.319	n.a.	n.a.	n.a.
RN	rho	**0.623**	0.6	−0.084	**0.625**	0.476	0.126	n.a.	n.a.	n.a.
	*P*	**<0.001**	0.051	0.795	**<0.001**	0.118	0.697	n.a.	n.a.	n.a.
STN	rho	−0.268	0.309	−0.182	**0.468**	0.147	0.21	n.a.	n.a.	n.a.
	*P*	0.206	0.355	0.571	**0.012**	0.649	0.513	n.a.	n.a.	n.a.
ND	rho	0.296	**0.594**	0.056	0.098	0.517	0.343	n.a.	n.a.	n.a.
	*P*	0.15	**0.042**	0.863	0.633	0.085	0.276	n.a.	n.a.	n.a.

*rho, Spearman correlation coefficient; P, significance (P-values < 0.05 are printed bold); THAL, Thalamus; CAU, caudate; PUT, Putamen; PAL, Pallidum; HIP, Hippocampus; AMY, Amygdala; NAC, Accumbens; SN, Substantia Nigra; RN, Red Nuclues; STN, Subthalamic Nucleus; ND, Dentate Nucleus; n.a., not acquired*.

To account for head size differences, all individual DGM volumes were normalized to the intracranial cavity volume (ICCV). The intracranial cavity volumes (ICCV) were determined for each subject using NeuroQLab software tools (Fraunhofer-Mevis, Bremen Germany) (3).

### Statistical Analysis

Statistical analyses were performed using SPSS24 (IBM SPSS, Chicago, USA). Parametric statistical tests were used for the linear scaled variables which fulfilled the condition of normal distribution (Kolmogorov-Smirnov tests), like the MRI results, age, and disease duration. Non-parametrical tests were applied when ordinal variables like disability scores and neuropsychological test results were investigated.

For all DGM nuclei in the healthy control group, the dependence of susceptibility, R2^*^ and volume on physiological aging was investigated using Pearson's correlation analyses.

Susceptibility, R2^*^and DGM volumes were reported as mean values and standard deviation in the patient groups and in healthy controls. To account for disease related age differences between the patient groups, age matched sub-groups of the healthy controls were selected and compared separately to the corresponding patient group by univariate analysis of variance (ANOVA) of each variable including age as a covariate and group as fixed factor.

Inter-group comparisons between the three patient subgroups (DM1, DM2, DM1_c.o._) regarding R2^*^, DGM volumes, age, and disease duration were performed utilizing univariate ANOVA with subsequent *post-hoc* pairwise tests adjusted for multiple comparisons using Games-Howell correction.

If these pairwise differences were significant when comparing patient subgroups and matched healthy controls, then the effect size estimates of the differences were calculated using Cohen's *d* based on the mean and standard deviation and group sizes. Effect sizes are interpreted as small when *d* ≤ 0.3, medium when 0.3 < *d* ≤ 0.5, and strong when *d* > 0.5 (58).

Inter-group differences between disability scores and neuropsychological test results were assessed using Kruskal-Wallis tests with *post-hoc* pairwise Dunn's tests adjusted for multiple comparisons using Bonferroni correction.

Spearman correlation analyses were used to assess associations between disability scores, demographical, and MRI data.

## Results

### Susceptibility, R2^*^ and Volumes of DGM

#### Physiological Aging Effects in Healthy Controls

We detected physiological age dependency in the healthy control group for susceptibility in the putamen, pallidum, the substantia nigra, and red nucleus; R2^*^ was age dependent in the control group in the thalamus, putamen, the substantia nigra, and subthalamic nucleus. These findings are in accordance with the well-established increase of iron content in these structures with normal aging (59), indicating that sources of susceptibility and R2^*^ changes are dominated by increases in iron content in DGM. DGM volumes in the control group were significantly negatively correlated with age in the thalamus, caudate nucleus, putamen, and pallidum. Details of the physiological age dependencies of susceptibility, R2^*^ and DGM volumes in the healthy control group, as well as the age dependency in the patient groups are provided in Table 3.

Because of the age dependency of susceptibility, R2^*^ and DGM volumes we selected age matched sub-groups of the healthy controls corresponding to each patient group when comparing the susceptibility, R2^*^ and volumes between the patients and controls.

#### Inter-Group Differences

**Figure 2:**
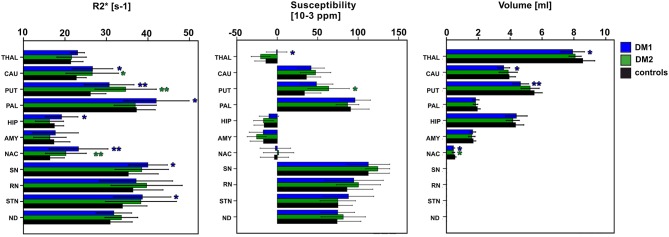
Group comparisons between classical DM1, DM2, and healthy controls (entire control group) for DGM nuclei: THAL, thalamus; CAUD, caudate; PUT, putamen; PAL, pallidum; HIP, hippocampus; AMY, amygdala; NAC, N. accumbens; SN, substantia nigra; RN, red nucleus; STN, subthalamic nucleus; ND, dentate nucleus. Boxes, error bars: mean ± 1 standard deviation. Significance of group differences compared to age matched healthy controls: ^*^P < 0.050, ^**^P < 0.010.

Group comparisons of R2^*^, susceptibilities and DGM volumes between DM1, DM1_c.o._, DM2, and age matched controls are illustrated as bar plots in Figure 2 and summarized in detail in Tables 4–6. In healthy controls, R2^*^ and susceptibility were high in pallidum and other known iron-rich structures like substantia nigra, red nucleus, subthalamic nucleus and dentate nucleus and lower in the hippocamus, amygdala, accumbens, caudate nucleus, putamen, and the thalamus.

**Table 4:** Group comparisons of the transverse relaxation rate R2^*^between DM1, DM1c.o., DM2, and age matched controls (HC).

Transverse relaxation rate R2* **[s**^**−1**^**]**														
**DGM structure**	**DM1** ***N*** **=** **12 Mean (SD)**	**HC**_**DM1**_ ***N*** **=** **24 Mean (SD)**	***P*****-valueDM1**^**a**^	**Effect size**^**b**^	**DM2** ***N*** **=** **12 Mean (SD)**	**HC**_**DM2**_ ***N*** **=** **16 Mean (SD)**	***P*****-valueDM2**^**a**^	**Effect size**^**b**^	**DM1**_**c.o.**_ ***N*** **=** **4 Mean (SD)**	**HC**_**DM1c.o.**_ ***N*** **=** **11 Mean (SD)**	***P*****-valueDM1**_**c.o.**_^**a**^	***P*****-valueDM1**_**c.o.**_**-DM1**^**d**^	***P*****-valueDM1**_**c.o.**_**-DM2**^**e**^	***P*****-value between patient groups**^**c**^
THAL	23.1 (1.6)	21.4 (2.5)	0.078	–	21.6 (3.5)	22.3 (2.9)	n.s.	–	22.0 (2.4)	20.0 (2.9)	n.s.	n.s.	n.s.	n.s.
CAU	**25.8 (5.5)**	**22.8 (2.2)**	**0.037**	0.72	26.6 (6.3)	23.4 (2.4)	0.079		**17.4 (0.7)**	**22.8 (2.3)**	**0.003**	0.002	0.001	0.028
PUT	**30.7 (6.)**	**26. (2.7)**	**0.001**	1.01	**34.6 (7.4)**	**28.3 (3.3)**	**0.006**	1.10	20.9 (1.6)	23.1 (2.1)	n.s.	0.001	<0.001	0.004
PAL	**42. (7.9)**	**37.3 (4.3)**	**0.041**	0.74	36.9 (5.1)	38.6 (4.1)	n.s.	–	35.4 (2.9)	34.6 (3.9)	n.s.	n.s.	n.s.	n.s.
HIP	**19.2 (3.9)**	**17.1 (1.8)**	**0.036**	0.69	16.3 (3.4)	17.4 (2.5)	n.s.	–	**14.4 (3.3)**	**17.6 (1.4)**	**0.034**	n.s.	n.s.	0.088
AMY	17.7 (5.5)	17.0 (3.5)	n.s.	–	16.4 (3.9)	17.5 (4.6)	n.s.	–	15.3 (4.9)	17. (2.7)	n.s.	n.s.	n.s.	n.s.
NAC	**23.3 (7.1)**	**16.7 (3.3)**	**0.001**	1.19	**20.2 (4.9)**	**15.8 (3.9)**	**0.013**	0.99	**12.1 (1.9)**	**17.5 (2.8)**	**0.003**	0.001	<0.001	0.013
SN	**40.0 (4.7)**	**35.1 (6.6)**	**0.044**	0.86	38.5 (6.4)	38. (6.7)	n.s.	–	32.3 (4.0)	30.1 (5.6)	n.s.	0.046	n.s.	0.069
RN	37.1 (8.8)	36.6 (6.4)	n.s.	–	39.7 (8.6)	40. (5.2)	n.s.	–	27.1 (4.5)	30.0 (5.1)	n.s.	0.033	0.009	0.047
STN	**38.7 (6.9)**	**33.6 (5.1)**	**0.025**	0.84	38.3 (8.6)	35.5 (5.4)	n.s.	–	33.1 (4.7)	30.7 (5.4)	n.s.	n.s.	n.s.	0.002
ND	31.8 (4.3)	31.2 (5.)	n.s.	–	33.6 (4.0)	32.0 (5.2)	n.s.	–	24.7 (2.1)	29. (4.8)	n.s.	0.005	<0.001	n.s.

*Bold values indicate significant paired group differences; effect size (Cohen's d) presented for significant pairwise group differences in DM1 and DM2*.

*DGM, deep gray matter; DM, Myotonic dystrophy, HC, healthy controls; N, number of subjects; SD, standard deviation; THAL, thalamus; CAU, caudate; PUT, putamen; PAL, pallidum; HIP, hippocampus; AMY, amygdala; NAC, accumbens; SN, substantia nigra; RN, red nucleus; STN, subthalamic nucleus; ND, dentate*.

a *Significance of univariate ANOVA including age as covariate of pairwise comparisons between patient group and matched HC subgroup (P-values < 0.05 are printed bold; n.s., not significant with P-value > 0.2)*.

b *Absolute effect size: Cohen's d is shown for significant group differences if P-values < 0.05*.

c *Significance of univariate ANOVA: differences between patient groups (DM1, DM2, DM1_c.o_)*.

**Table 5:** Group comparisons of the magnetic susceptibility (relative to CSF in anterior lateral ventricles) between DM1, DM2 and age matched controls (HC); in DM1_c.o_. and HC_DM1c.o._ the susceptibility is referenced relative to whole brain WM.

**Magnetic susceptibility/10**^****−3****^ **ppm**												
**DGM structure**	**DM1** ***N*** **=** **12 Mean (SD)**	**HC**_**DM1**_ ***N*** **=** **24 Mean (SD)**	***P*****-valueDM1**^**a**^	**Effect size**^**b**^	**DM2** ***N*** **=** **12 Mean (SD)**	**HC**_**DM2**_ ***N*** **=** **16 Mean (SD)**	***P*****-value DM2**^**a**^	**Effect size**^**b**^	**DM1**_**c.o.**_^**c**^ ***N*** **=** **4 Mean (SD)**	**HC**_**DM1c.o.**_^**c**^ ***N*** **=** **11 Mean (SD)**	***P*****-valueDM1**_**c.o**_^**a**^	***P*****-valueDM1-DM2**^**a**^
THAL	**−0.9 (12.7)**	**−11.5 (13.6)**	**0.039**	**0.81**	−20.6 (11.7)	−15.7 (11.4)	n.s.	–	−0.44 (2.95)	0.49 (4.2)	n.s.	**<0.001**
CAU	41.7 (16.6)	40.4 (16.0)	n.s.	–	47.3 (19.)	38.6 (15.0)	0.192	–	**27.2 (5.7)**	**44.0 (6.2)**	**<0.001**	n.s.
PUT	48.5 (20.3)	37. (19.1)	0.179	–	**63.5 (25.6)**	**41.9 (16.9)**	**0.014**	**1.0**	27.5 (4.9)	34.3 (7.7)	0.149	n.s.
PAL	96.0 (19.1)	94.6 (22.1)	n.s.	–	86.8 (14.1)	97.8 (22.1)	0.155	–	96.3 (10.)	93. (14.)	n.s.	0.196
HIP	−10.3 (12.1)	−13.8 (12.5)	n.s.	–	−17.0 (12.2)	−18.1 (8.5)	n.s.	–	−3.9 (2.7)	−1.8 (4.9)	n.s.	0.086
AMY	−17.4 (17.3)	−14.1 (16.0)	n.s.	–	−25.5 (11.7)	−19. (13.9)	0.180	–	−2.4 (7.6)	−3.5 (11.5)	n.s.	0.110
NAC	−2.4 (18.9)	−1.0 (18.2)	n.s.	–	1.7 (18.7)	−1.8 (18.9)	n.s.	–	13.7 (15.1)	6.4 (16.9)	n.s.	n.s.
SN	112.6 (26.4)	118.7 (22.8)	n.s.	–	124.2 (14.8)	120.7 (20.6)	n.s.	–	**95.8 (25.7)**	**127.7 (16.7)**	**0.023**	n.s.
RN	94.6 (36.5)	91.7 (26.9)	n.s.	–	100.3 (27.4)	100.7 (16.7)	n.s.	–	**61.1 (14.6)**	**90.5 (22.7)**	**0.042**	n.s.
STN	87.9 (31.3)	75.4 (18.3)	0.171	–	74.8 (21.9)	71.9 (15.7)	n.s.	–	85.9 (24.8)	85.9 (23.1)	n.s.	0.100
ND	74.7 (20.9)	73.2 (29.)	n.s.	–	81.5 (27.5)	77.7 (28.5)	n.s.	–	70.6 (7.0)	83.4 (19.3)	n.s.	n.s.
CSF^c^	10.2 (7.6)	10.9 (11.9)	n.s.	–	15.0 (9.2)	14.4 (9.7)	n.s.	–	**1.2 (9.1)**	**16.4 (12.8)**	**0.031**	n.s.

*Bold values indicate significant paired group differences; effect size (Cohen's d) presented for significant pairwise group differences in DM1 and DM2*.

*DGM, deep gray matter; DM, Myotonic dystrophy; HC, healthy controls; N, number of subjects; SD, standard deviation; THAL, thalamus; CAU, caudate; PUT, putamen; PAL, pallidum; HIP, hippocampus; AMY, amygdala; NAC, accumbens; SN, substantia nigra; RN, red nucleus; STN, subthalamic nucleus; ND, dentate; CSF, cerebrospinal fluid measured within anterior ventricular horns*.

a *Significance of univariate ANOVA including age as covariate of pairwise comparisons between patient group and matched HC subgroup (P-values < 0.05 are printed bold; n.s., not significant with P-value > 0.2)*.

b *Absolute effect size: Cohen's d is shown for significant group differences if P-values < 0.05*.

c *Magn. Susceptibility [ppm] was referenced to whole brain WM for childhood onset DM1; in all other analyses: referenced to cerebrospinal fluid measured within anterior ventricular horns*.

**Table 6:** Group comparisons of DGM volumes between DM1, DM1_c.o_., DM2, and age matched controls (HC).

**DGM volume/ml**												
**DGM structure**	**DM1** ***N*** **=** **12 Mean (SD)**	**HC**_**DM1**_ ***N*** **=** **24 Mean (SD)**	***P*****-valueDM1**^**a**^	**Effect size**^**b**^	**DM2** ***N*** **=** **12 Mean (SD)**	**HC**_**DM2**_ ***N*** **=** **16 Mean (SD)**	***P*****-value DM2**^**a**^	**Effect size**^**b**^	**DM1**_**c.o.**_ ***N*** **=** **4 Mean (SD)**	**HC**_**DM1c.o.**_ ***N*** **=** **11 Mean (SD)**	***P*****-value DM1**_**c.o.**_^**a**^	***P*****-value between patient groups**^**c**^
THAL	**7.92 (0.74)**	**8.54 (0.76)**	**0.038**^**a**^	−0.83	8.08 (0.39)	8.22 (0.53)	n.s.	–	8.48 (0.27)	8.86 (0.61)	n.s.	
CAU	**3.59 (0.38)**	**3.88 (0.34)**	**0.048**^**a**^	−0.84	3.88 (0.57)	3.71 (0.26)	n.s.	–	4.06 (0.55)	4.20 (0.37)	n.s.	
PUT	**4.65(0.51)**	**5.51 (0.52)**	**<0.001**^**a**^ **0.006**^**e**^	−1.67	5.25 (0.57)	5.21 (0.38)	n.s.	–	5.88 (0.84)	5.84 (0.42)	n.s.	0.003
PAL	1.83 (0.22)	1.94 (0.16)	n.s.	–	1.83 (0.14)	1.86 (0.15)	n.s.	–	2.01 (0.08)	2.04 (0.13)	n.s.	
HIP	4.39 (0.69)	4.34 (0.56)	n.s.	–	4.17 (0.41)	4.24 (0.49)	n.s.	–	4.29 (0.25)	4.35 (0.49)	n.s.	
AMY	1.65 (0.19)	1.67 (0.18)	n.s.	–	1.59 (0.21)	1.62 (0.13)	n.s.	–	1.70 (0.11)	1.70 (0.11)	n.s.	
NAC	**0.44 (0.08)**	**0.52 (0.07)**	**0.010**^**a**^	−1.06	**0.44 (0.08)**	**0.52 (0.08)**	**0.012**^**a**^	1.0	0.54 (0.05)	0.54 (0.08)	n.s.^a^ **0.043**^d^ **0.034**^e^	0.061

*Bold values indicate significant paired group differences; effect size (Cohen's d) presented for significant pairwise group differences in DM1 and DM2*.

*DGM, deep gray matter; DM, Myotonic dystrophy; HC, healthy controls; N, number of subjects; SD, standard deviation; THAL, thalamus; CAU, caudate; PUT, putamen; PAL, pallidum; HIP, hippocampus; AMY, amygdala; NAC, accumbens*.

a *Significance of univariate ANOVA including age as covariate of pairwise comparisons between patient group and matched HC subgroup (P-values < 0.05 are printed bold; n.s., not significant with P-value > 0.2)*.

b *Absolute effect size: Cohen's d is shown for significant group differences if P-values < 0.05*.

c *Significance of univariate ANOVA: differences between patient groups (DM1, DM2, DM1_c.o._)*.

^d,e^ Post-hoc pairwise tests adjusted for multiple comparisons using Games-Howell correction:

d Compared to DM1;

e *Compared to DM2*.

For DM1 patients, R2^*^ was significantly higher compared to healthy controls especially in the putamen and accumbens, but also in the caudate nucleus, pallidum, hippocampus, substantia nigra, and subthalamic nucleus. The effect sizes (Cohen's *d*) of these differences were high (0.69–1.19), indicating strong effects. In contrast, susceptibility in the putamen was slightly higher in DM1 than in controls but did not reach statistical significance. No other susceptibility differences were significant in DGM structures in which significant R2^*^ alterations between DM1 and controls have been observed.

While for R2^*^ in the thalamus only a tendency toward higher values in patients has been observed, significantly higher susceptibilities in DM1 patients compared to controls were determined in the thalamus.

Similar to DM1, DM2 patients showed significantly elevated R2^*^ in putamen and accumbens, with high effect sizes of 1.10 and 0.99, respectively. There was a trend toward higher R2^*^ in caudate nucleus of DM2 compared to controls, which was reinforced by higher—although not significant—susceptibility in this structure. Differences in the susceptibility between DM2 and controls were significant merely in the putamen, where the mean susceptibility as well as the intra-group variability (standard deviation) were considerably higher in the patient group.

We detected only few significant differences between the DM1 and DM2 patients. The thalamus susceptibility was higher in DM1 than in DM2. The susceptibility in the putamen was markedly higher in the DM2 group than in DM1, but the difference did not reach statistical significance (*p* = 0.056).

Although the patient number in the DM1_c.o._ group was small, we exploratorily studied alterations in this patient group focusing on differences with *P*-values < 0.010. We detected significantly smaller values of R2^*^ in the caudate nucleus and accumbens in comparison to the age matched controls (*P* = 0.001) and compared to DM1 or DM2 (*P* < 0.001), which was in opposition to the alterations found in the group comparison between DM1 and DM2 groups and controls, where R2^*^ increases had been observed in the patient groups. A similar decrease compared to controls was observed for the susceptibility of the caudate nucleus in DM1_c.o._ (*P* < 0.001). Conventional MRI imaging showed no obvious structural abnormalities or brain atrophy in these patients with childhood-onset DM1. Exemplary MRI images are provided in the supplement (Figure S1).

The volumes of putamen, accumbens, and to a lesser extent of thalamus and caudate nucleus were significantly reduced in DM1 patients compared to healthy controls. In the DM2 group we detected significant volume reductions only in the accumbens compared to healthy controls. In contrast, no volume loss was found in any investigated DGM structure in DM1_c.o._ patients compared to healthy controls.

### Associations Between DGM Atrophy and Susceptibility or R2^*^

Prior to the correlation analyses between MRI results, the potential age-dependencies of all MRI results in each patient group were assessed (Table 3). Similar to the findings in healthy controls, susceptibility increased significantly with age in caudate nucleus, putamen, accumbens and dentate nucleus in DM1. R2^*^ was positively correlated with age in putamen, while caudate nucleus volume was inverse correlated with age in DM1. In the DM2 group, susceptibility and R2^*^ depended on age in caudate nucleus. Additionally, R2^*^ and volume of thalamus were inversely correlated with age in DM2. Correlations between the age-dependent structures were assessed by partial correlation analyses controlled for age, while the other combinations were investigated by Spearman correlation analyses.

In DM1, caudate nucleus volume was inversely correlated with R2^*^ of caudate nucleus (correlation coefficient/significance: rho/*P* = −0.818/0.001). Interestingly, R2^*^ and susceptibility alterations in putamen, which were highly significant compared to healthy controls, were not correlated to atrophy in the putamen or any other DGM structure in DM1. This indicated that independent pathophysiological mechanisms may be involved in these effects.

In contrast to DM1 no significant correlations between caudate nucleus atrophy and alterations in susceptibility or R2^*^ were observed in DM2.

In the healthy control group, we detected no significant correlations between DGM volumes and susceptibility or R2^*^ in these structures.

### Clinical and Neuropsychological Results

The degree of muscular impairment as assessed by the MIRS was higher in DM1 compared to childhood-onset DM1_c.o._ and compared to the adapted MIRS in DM2 (Table 1). MIRS correlated significantly with disease duration in DM1 (Spearman's correlation coefficient rho/*P*: 0.785/0.002), indicating a progressive course of muscular impairment in this patient group.

Daytime sleepiness, which was assessed by the ESS, was more frequent and more severe (ESS > 11) with a percentage of 58% patients in the DM1 group compared to 25% in DM1_c.o._ and 20% in DM2 patients (Table 1).

The mean depression score was higher in DM2 than in the two DM1 subgroups (Table 1). No participant received antidepressant therapy during the study.

In the DM2 group, 58% of the patients showed additional Parkinsonian or cerebellar symptoms like extrapyramidal rigidity dysmetria or restless-legs-syndrome, while only one patient in the DM1 group suffered from rigor.

Cognitive deficits in the DM1 and the DM2 patient groups were merely found in selective tasks, affecting mainly verbal and non-verbal short-term memory, attention and flexibility of thinking, while no reduction of the long-term verbal memory and general intelligence level was observed. The degree of cognitive impairment was similar in DM1 and DM2 with a tendency for worse performance in executive functions (verbal fluency with change of categories) in the DM1 group. In the DM1_c.o._ patients, no major cognitive impairment was reflected by the neuropsychological test results. Table 2 summarizes these findings: results are shown for those tests in which at least 25% of patients in one of the groups had abnormal results.

### Associations Between MRI-Derived Parameters and the Clinical and Neuro-Cognitive Status

Since none of the clinical parameters, muscular weakness, daytime sleepiness or depression, was significantly correlated with age, Spearman correlation analyses were chosen to assess associations between these variables and MRI outcomes. Since the CTG repeat length in DM1 correlated negatively with age (rho/*P*: −0.610/0.035), correlation analyses of the CTG repeat length with the MRI-derived parameters were controlled for age (partial correlations).

### Muscular Impairment (MIRS)

In DM1, we found a negative correlation between the MIRS and thalamus volume (rho/*P*: −0.721/0.008), putamen volume (rho/*P*: −0.583/0.046) and accumbens volume (rho/*P*: −0.638/0.025), relating more severe muscular weakness to higher grades of DGM atrophy in these structures. Furthermore, caudate nucleus susceptibility was significantly correlated with MIRS in DM1 (rho/*P*: 0.674/0.016).

### Daytime Sleepiness (ESS)

**Figure 3:**
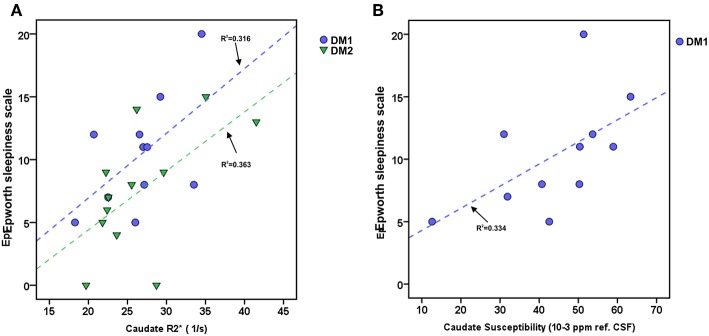
Associations between daytime sleepiness (ESS) and Caudate R2^*^ or Caudate susceptibility in DM1 and DM2. **(A)** Scatterplot of ESS and Caudate R2^*^ in DM1 (blue) and DM2 (green). **(B)** Scatterplot of ESS and Caudate susceptibility (unit: ppm referenced to CSF) in DM. Dashed lines: linear regression (R^2^ = regression coefficient).

The ESS of DM1 patients correlated significantly with the caudate nucleus susceptibility (rho/*P*: 0.633/0.037) and in tendency with caudate nucleus R2^*^ (rho/*P*: 0.537/0.089). We found no significant associations between DGM volume loss and ESS in this patient group. In the DM2 group, ESS correlated significantly with caudate nucleus R2^*^ (rho/*P*: 0.586/0.045) (Details are shown in Figure 3).

### Depression

In DM2, the depression score was significantly correlated with caudate nucleus susceptibility (rho/*P*: 0.629/0.028), with putamen volume reduction (rho/*P*: −0.625/0.030), and in tendency with volume loss of the hippocampus (rho/*P*: −0.544/0.067).

Since in DM1 most of the patients had no clinically manifest depression, correlation analyses were omitted in this group.

### CTG Repeat Length

We observed no significant correlations between the CTG repeat length in DM1 patients and susceptibility, R2^*^or DGM volumes, respectively.

### Associations of Susceptibility, R2^*^, and DGM Volumes With Neuropsychological Scores

When comparing patients with normal neuropsychological test results with those who had cognitive deficits, we found higher R2^*^ in the hippocampus of DM1 patients with abnormal tonic and phasic alertness (Table 7). Furthermore, in this group abnormal results in flexibility of thinking (executive functions: verbal fluency, categorical) were related with higher R2^*^ of caudate nucleus and accumbens, and higher susceptibility of the hippocampus. Interestingly, DM1 patients with impaired verbal fluency (categorical) had significantly smaller thalamus volumes than patients with normal neuropsychological test results.

**Table 7:** Group comparison of R2^*^ [s^−1^], magnetic susceptibility [10^−3^ ppm referenced to CSF] and volumes [ml] of the DGM structures between patients with normal and abnormal neuropsychological test results: only significant results with *P* < 0.050 are reported.

**Group**	**Test**	**DGM structure**		**Normal**	**Abnormal**	***P*^**a**^**
DM1	Tonic alertness	HIP R2^*^	*N* (%)	5 (42%)	7 (58%)	0.010
			Median	16.4	20.4	
			[Min.; Max.]	[13.3; 19.2]	[17.2; 28.4]	
	Phasic alertness	HIP R2^*^	*N* (%)	5 (42%)	7 (58%)	0.010
			Median	16.4	20.4	
			[Min.; Max.]	[13.3; 19.2]	[17.2; 28.4]	
	Executive functions: alternating category verbal fluency tasks	CAU R2^*^	*N* (%)	4 (33%)	8 (66%)	0.016
			Median	19.5	27.3	
			[Min.; Max.]	[16.8; 26.5]	[22.5; 34.5]	
		NAC R2^*^	*N* (%)	4	8	0.008
			Median	14.0	24.6	
			[Min.; Max.]	[13.4; 22.6]	[20.7; 34.8]	
		HIP susceptibility	*N* (%)	4 (33%)	8 (66%)	0.016
			Median	−2.0	3.0	
			[Min.; Max.]	[−13; 3]	[−8; 11]	
		THAL Vol.	*N* (%)	4 (33%)	8 (66%)	0.028
			Median	8.2	7.7	
			[Min.; Max.]	[7.8; 9.6]	[6.7; 8.8]	
DM2	Tonic alertness	STN susceptibility	*N* (%)	5 (42%)	7 (58%)	0.002
			Median	63.5	81.5	
			[Min.; Max.]	[54.6; 64.3]	[66.1; 134.2]	
	CNS symptoms Parkinsonian/cerebellar/RLS	SN susceptibility	*N* (%)	5 (42%)	7 (58%)	0.030
			Median	117.8	134.0	
			[Min.; Max.]	[96.7; 121.2]	[105.4; 145.4]	

N (%), number and percentage of subjects with pathological or normal test results; DGM, deep gray matter; min., minimum; max., maximum; vol., volume; R2^*^, Transverse relaxation rate [s^−1^]; HIP, hippocampus; THAL, thalamus; CAU, caudate; NAC, N. accumbens; SN, substantia nigra.

a *P-value: significance of group comparison (Mann-Whitney-U test) between normal and pathological test results*.

In the DM2 group, impaired tonic alertness was related with significantly higher susceptibility in the subthalamic nucleus. Additionally, the presence of CNS symptoms [Parkinsonian, cerebellar, or restless legs symptoms (RLS)] was associated with significantly higher susceptibility in the substantia nigra.

We found no associations of susceptibility, R2^*^ and DGM volumes with any of the other tests referred to in Table 2 (divided attention, verbal short term memory, non-verbal short term memory).

## Discussion

In the present study, we used quantitative mapping of the susceptibility and the effective transverse relaxation rate as indirect measures of iron content in DGM in DM1 and DM2 patients in comparison to healthy controls. MRI findings indicative of increased iron content (elevation mainly of R2^*^ and partly of the susceptibility) were found in DM1 and DM2 with most pronounced changes in the putamen and accumbens (Figure 2, Tables 4, 5). In contrast to DM2, additional significant affection of DGM structures (caudate, pallidum, hippocampus, substantia nigra and subthalamic nucleus) by R2^*^ increase was detected in DM1. Thus, taking into account that the disease duration was similar in both groups, DGM iron accumulation seems to be a more widespread phenomenon in DM1 than in DM2.

The effect sizes of these DGM alterations were high (Cohen's *d* between 0.99 and 1.10 for putamen and accumbens; see Tables 4, 5). In comparison, in a recent study using the same methodology for patients with Parkinson's disease were iron accumulation in the substantia nigra is a well-known effect, the effect size of R2^*^ increase in the substantia nigra was 0.5 (60). Thus, iron accumulation in DGM structures in myotonic dystrophies may indeed be interpreted as a substantial effect although its cause is unclear so far. It maybe speculated that misfolding of proteins, neurofibrillary tangles, and micro-RNA deposits which accumulate in specific brain tissues may play a pathogenic role (61): In DM1 autopsy studies neuronal cytoplasmic inclusion bodies in the thalamus and in the caudate nucleus have been described (62, 63). Brain tau pathology with neurofibrillary tangles was detected in the hippocampus in DM1 and DM2 and in the substantia nigra in DM2 (64). These disease related tissue alterations could be attributed to impose oxidative stress on cellular structures. On the other hand, DGM nuclei possess the highest iron content also in the healthy human brain, increasing physiologically with normal aging (59). Due to the genetic defect DGM structures in DM1 and DM2 may be especially susceptible to disease related cellular damage and consecutive premature aging effects (65). In addition, striatal cellular repair mechanisms have been shown to be less effective in autopsy studies in single Huntington disease patients compared to other regions in brain, such as the cerebellum suggesting structural peculiarities regarding compensation of repeat associated pathology (66). Overall, in our study the relaxation rate R2^*^ seemed to be the more sensitive method for detecting subtle group differences than susceptibility. This finding may seem contradictory to other studies on patients with Parkinson's disease or Multiple Sclerosis in which a higher sensitivity of the susceptibility due to additional effects of iron accumulation and demyelination has been reported (28, 60). One reason might be that in our study the susceptibility in patients and controls was more variable in all investigated structures. Accordingly, in other recent studies (60, 67) higher inter-subject variability in susceptibility compared to R2^*^ in healthy controls and in Parkinson disease has been observed. Furthermore, susceptibility is not an absolute quantity, but has to be referenced relative to a different region in the brain in which the susceptibility is stable and unaffected by disease related alterations (for example: ventricular CSF, frontal WM, or whole brain susceptibility). The intrinsic fluctuations in this reference region may additionally contribute to the variability of the magnetic susceptibility. Recent studies elucidated this problem in detail (67, 68). Differences in the myelin content of specific DGM tissues in Myotonic Dystrophy could be another confounding effect (27).

Interestingly, when regarding the DM1_c.o._ patients, we found opposite alterations in some DGM structures compared to the effects in DM1, mainly R2^*^ reduction in caudate nucleus, accumbens and hippocampus. The pathophysiological basis of these effects is still unclear. We hypothesize that the relatively young age of these patients compared to the other groups, makes neuro-developmental effects more likely than neurodegeneration as sources of these alterations. Lower iron concentrations or disturbed brain iron homeostasis, dysmyelination or a higher interstitial water content in the affected structures as results of neuro-developmental disturbances might be reasons for the observed reduced relaxation rates and susceptibility in DM_c.o._ patients (69).

### DGM Atrophy in Patients

Although disease duration and age were similar in the DM1 and the DM2 groups, more DGM structures were affected by atrophy in DM1 (putamen, accumbens, thalamus, and caudate) than in DM2 (accumbens only), supporting the hypothesis based on the R2^*^ and susceptibility findings, that DGM structures are a specific target of disease related alterations in DM1. The same structures in which atrophy was found in DM1 were also prone to an age related volume decrease in healthy controls, indicating that aging processes might be accelerated in DM1 in these DGM structures. In contrast, accumbens atrophy in DM1 and DM2, which was not paralleled by age dependent volume loss in healthy controls, is probably evoked by other pathophysiological mechanisms. Furthermore, significant associations in DM1 between accumbens atrophy and R2^*^ elevation gives rise to the hypothesis that neurodegeneration, secondary to increased iron concentrations, might be a source of atrophy in accumbens in this disease entity (70).

### Associations Between DGM Alterations and Neurocognitive Performance, Daytime Sleepiness, and Depression

Among the selective cognitive deficits which were found in DM1 and DM2 patients (Table 2) merely alertness and executive functions assessed by alternating category verbal fluency tasks were associated with DGM alterations representing iron accumulation or volume loss. Furthermore, DM2 patients who showed additional CNS symptoms had increased SN susceptibility, while daytime sleepiness and depression were correlated with iron accumulation in the caudate nucleus. Bearing in mind the exploratory character of the present study and the limited number of patients, hypotheses about the pathophysiological or even causal relations between tissue iron levels and impairment of neurofunctional networks in the brain can only be speculative. Nevertheless, it can be assumed that dysfunction of DGM which are relevant key structures within the neuronal networks and the brain connectome could also contribute to cognitive impairment—besides the probably more relevant affection of cortical structures as shown in previous studies [for example (3, 13)].

In the following section, we set up preliminary hypotheses about potential interrelations between DGM iron accumulation and cognitive functions:

Correlation analyses of group differences revealed that DM1 and DM2 patients with or without impaired alertness differed according to R2^*^ and susceptibility elevations in hippocampus (DM1) and subthalamic nucleus (DM2). Both structures are thought to be functionally involved in attention and alertness: the hippocampus, as a component of the limbic system, does not only play a major role in memory processing, but is also involved in attentional control via reciprocal connections with the prefrontal cortex (71). Similarly, apart from its classical assignment to the motor system, the subthalamic nucleus is involved in prefrontal and limbic functional loops which drive alertness and motivation control (72).

In our study, executive dysfunction in DM1 was associated with alterations involving multiple DGM nuclei, namely accumbens, caudate, hippocampus, and thalamus (Table 7). A common functional feature of these subcortical brain areas is their close link by frontostriatal functional loops to the frontal cortex, which is traditionally considered to be the major brain structure involved in executive functioning. These corticostrial executive networks involve most of the basal ganglia, namely caudate and accumbens, while striatal-cortical feedback is rendered through the thalamus (thalamostriatal loop) (73). Thus, iron accumulation in the involved DGM structures and thalamus atrophy might possibly constitute a part of the pathophysiological basis of executive impairment in DM1.

Findings in Parkinson's disease pointed to affection of motor control by increased iron content in the substantia nigra, due to its dopaminergic function (60). In analogy, in the present study occurrence of Parkinsonian or cerebellar symptoms or RLS, which were exclusively found in DM2 was associated with significantly elevated susceptibility in the substantia nigra, giving rise to the hypothesis, that substantia nigra impairment associated with increased iron content, might be a part of the symptom spectrum in DM2.

Day time sleepiness is a common and relevant non-muscular symptom in DMs and seems to be related to various factors including alterations of the sleep-wake cycle (74). In our groups of DM1 and DM2 patients, daytime sleepiness was significantly correlated with R2^*^ or susceptibility alterations in the caudate nucleus. Since the caudate nucleus is known to be involved in sleep regulation by frontostriatal and thalamostriatal networks, this correlation might possibly reflect a contribution of caudate nucleus iron accumulation to sleep dysregulation in DM1 and DM2 (75, 76).

Depression in DM2 was associated with volume loss in the hippocampus and putamen. These findings are in accordance with other morphometric studies, where specifically hippocampus atrophy has been described in patients with a major depression (77). As a new result we found a significant correlation between depression and caudate nucleus susceptibility suggesting a possible role of the caudate nucleus not only in motor control, executive functions and sleep regulation, but also in emotional processing. Iron accumulation could possibly lead to disturbances of reward-processing function of the caudate nucleus by direct tissue damage or by reducing the connectivity of the caudate with other involved structures like hippocampus or accumbens (78). Associations of depression have not been studied in DM1 since no clinically manifest depression has been detected in this group. This finding may have been biased by the phenomenon of anosognosia and unawareness of the disorder, which has been described as a common finding in DM1 patients with frontal cognitive impairment (79). Still, cognitive impairment was only mild and not indicative of major frontal dysfunction in our DM1 group.

## Limitations

The major limitation of our study is posed by the relatively small number of patients in each group which limits the statistical power of our findings. This might be justified by the explorative nature of the project, being the first study investigating iron accumulation in DGM in DMs. While the pathogenesis of iron accumulation in DM1 and DM2 brains is still unclear, our analyses pointed toward possible associations with clinical and cognitive functions. Still, we cannot draw conclusions from our cross-sectional analysis whether iron deposition is causative for neuronal degeneration or a concomitant effect. Further longitudinal studies would be needed to investigate course of iron accumulation in DM; alternatively studies on clinically very mildly affected patients could be helpful. Furthermore, the clinical relevance of the present findings needs to be confirmed and further elucidated in future studies including higher patient numbers.

Interpretation of the opposite findings concerning iron content in childhood onset and juvenile or adult onset patients remains speculative, given the small number of childhood-onset DM1 patients. Future studies with higher numbers of young DM1c.o. patients are warranted to gain more information to differentiate between developmental or degenerative disease processes.

Furthermore, a bias concerning the study population might be constituted by the study setting including mainly outpatients consulting the center for neuromuscular diseases who were able to tolerate a relatively long MRI examination. Thus, no patients with severe grades of motoric or cognitive impairment were included in the study. On the other hand, the relatively strong effect sizes of the observed DGM alterations even in moderately affected DM patients foster the hypothesis, that iron accumulation in the DM brain might be a pathophysiologically relevant effect.

A methodological limitation is given by the lack of a consensus about the strategy of referencing of the susceptibility (relative to CSF, brain white matter or other specific tissue types). This impedes the comparability of the susceptibility results of different studies.

## Summary and Conclusions

For the first time, specific and significant R2^*^ and susceptibility alterations representing increased iron accumulation in the basal ganglia and DGM nuclei have been detected in DM1 and DM2, with a more widespread involvement in DM1. While the pathophysiological basis and the clinical relevance is still unclear, these effects seemed to be associated with muscular impairment, depression, daytime sleepiness and specific cognitive deficits, showing a more widespread involvement of DGM structures in DM1.

Indications of opposed changes of R2^*^ or susceptibility have been found in several DGM structures in young, childhood-onset DM1_c.o._ patients compared to classical DM1.

Supported by these results, we speculate that iron accumulation which is regarded as a putative driving mechanism of tissue pathology in the brain, might have the potential to become a target of future therapeutic strategies in the myotonic dystrophies. Therefore, the presented finding should be further elucidated in future studies including larger patient groups.

## Data Availability Statement

The datasets generated for this study are available on request to the corresponding author.

## Ethics Statement

The studies involving human participants were reviewed and approved by the ethics committee of the Medical Faculty of the Ruhr-University Bochum (registration number 3794-10). The patients/participants provided their written informed consent to participate in this study.

## Author Contributions

SA: acquisition, analysis and interpretation of data, drafting and critical revision of the work for important intellectual content, and final approval of the version to be published. AD: analysis and interpretation of data, drafting and critical revision of the work for important intellectual content, and final approval of the version to be published. RS and CP: acquisition, analysis and interpretation of data for the work, and final approval of the version to be published. JR: interpretation of data, drafting and critical revision of the work for important intellectual content, and final approval of the version to be published. CL: conception, acquisition and interpretation of data for the work, critical revision of the work for important intellectual content, and final approval of the version to be published. CS-G and BB: design, acquisition, analysis, and interpretation of data for the work, drafting and critical revision of the work for important intellectual content, and final approval of version to be published and agreement to be accountable for all aspects of the work.

### Conflict of Interest

AD received a research grant by the German Research Foundation (DFG, DE 2516/1-1). CL received a research grant by the German Federal Ministry for Education and Research, BMBF, German Competence Network Multiple Sclerosis (KKNMS), grant no.01GI1601I, has received consulting and speaker's honoraria from Biogen Idec, Bayer Schering, Daiichi Sanykyo, Merck Serono, Novartis, Sanofi, Genzyme, and TEVA. CS-G has received a FoRUM grant (F 701-2010) from the Ruhr-University of Bochum and consulting and speaker's honoraria from Alexion Pharmaceuticals, Amicus Therapeutics, Bayer Schering, CSL Behring, Grünenthal, Lupin Pharmaceuticals, and TEVA. BB received financial support by the German Federal Ministry for Education and Research, BMBF, German Competence Network Multiple Sclerosis (KKNMS), grant no.01GI1601I. The remaining authors declare that the research was conducted in the absence of any commercial or financial relationships that could be construed as a potential conflict of interest.
