# Supplementary material for: Characterization of Iron Accumulation in Deep Gray Matter in Myotonic Dystrophy Type 1 and 2 Using Quantitative Susceptibility Mapping and R2* Relaxometry: A Magnetic Resonance Imaging Study at 3 Tesla
Source: Front Neurol. 2019 Dec 13;10:1320. doi: 10.3389/fneur.2019.01320 (PMC6923271; doi:10.3389/fneur.2019.01320)

Supplementary material to

Characterization of iron accumulation in deep grey matter in Myotonic Dystrophy type 1 and 2 using quantitative susceptibility mapping and R2* relaxometry: a magnetic resonance imaging study at 3 Tesla

Sevda Ates, Andreas Deistung, Ruth Schneider, Christian Prehn, Carsten Lukas[4], Jürgen R. Reichenbach, Christiane Schneider-Gold, Barbara Bellenberg

doi: 10.3389/fneur.2019.01320

Figure S1 Examples of a childhood DM1 (column a: age 25y, female, disease duration 15 y), a DM2 (column b: age 57y, female, disease duration 8 y) and a classical DM1 patient (column c: age 59y, female, disease duration 23 y) showing FLAIR weighted MRI images in axial, sagittal and coronal view. In contrast to classical adult DM1 and DM2 the childhood onset DM1 patient shows no signs of major brain atrophy and no obvious structural abnormalities

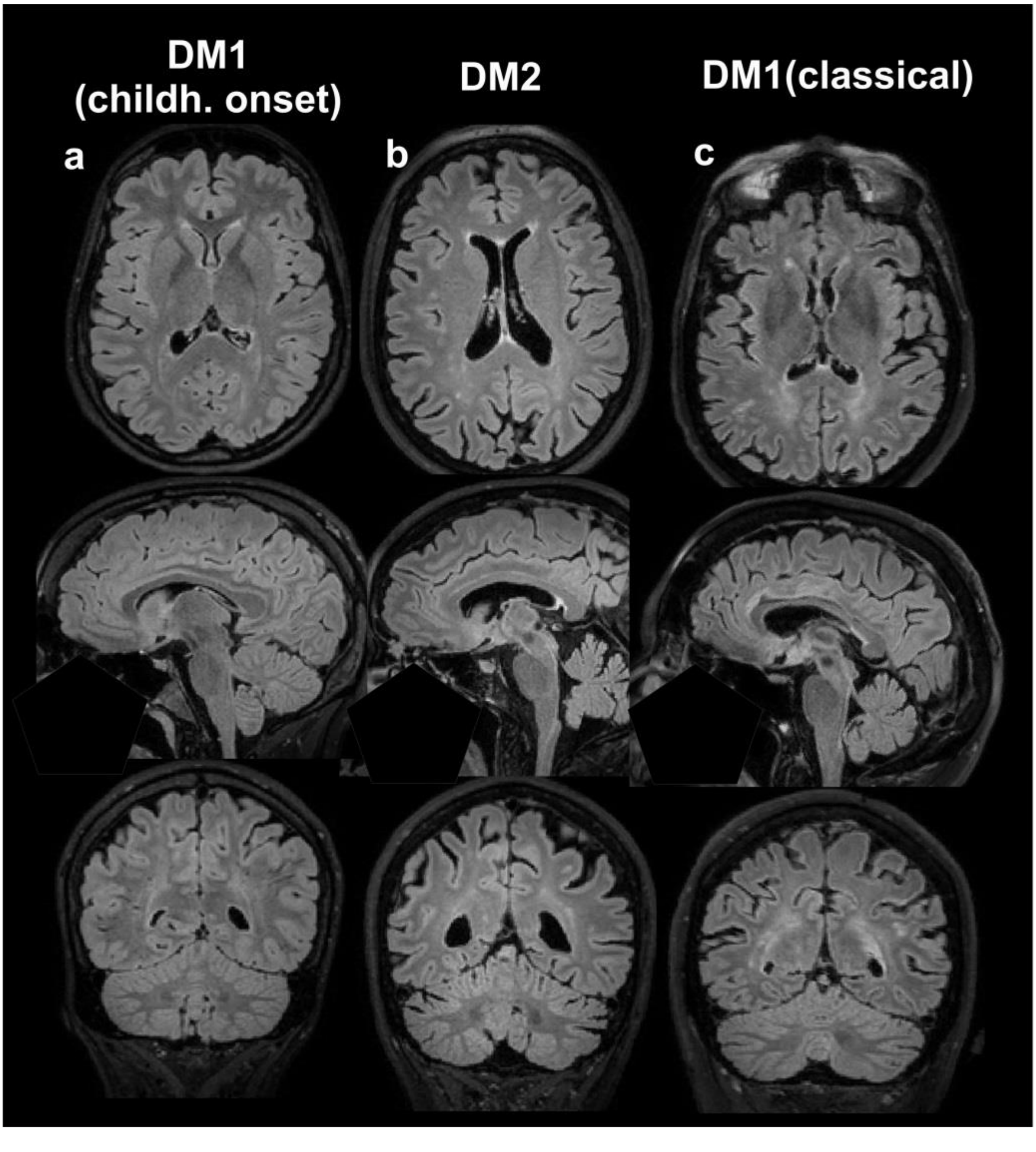

Supplement: Supplementary file 1 [file Presentation_1.pdf]
